# Radiation dose to staff from medical X-ray scatter in the orthopaedic theatre

**DOI:** 10.1007/s00590-023-03538-6

**Published:** 2023-04-01

**Authors:** T. Dorman, B. Drever, S. Plumridge, K. Gregory, M. Cooper, A. Roderick, E. Arruzza

**Affiliations:** 1 Jones Radiology, Eastwood, South Australia 5063 Australia; 2 SA Radiation, Adelaide, South Australia 5067 Australia; 3 Sportsmed, Stepney, South Australia 5069 Australia; 4 UniSA Clinical & Health Sciences, University of South Australia, Adelaide, South Australia 5000 Australia; 5 UniSA Allied Health & Human Performance, University of South Australia, Adelaide, South Australia 5000 Australia

**Keywords:** Radiation, Orthopaedics, Fluoroscopy, Nursing, Radiography

## Abstract

**Purpose:**

Given the growing demand for intraoperative imaging, there is increased concern for radiation dose for orthopaedic surgical staff. This study sought to determine the distribution of scatter radiation from fluoroscopic imaging in the orthopaedic surgical environment, with particular emphasis on the positions of personnel and the type of orthopaedic surgery performed.

**Methods:**

A radiation survey detector was deployed at various angles and distances around an anthropomorphic phantom. The scatter dose rate in microsieverts per hour (µSv/h) was recorded using consistent exposure parameters for five common surgical procedures. A C-arm unit produced radiation for the hip arthroscopy, hip replacement and knee simulations, whilst a mini C-arm unit produced fluoroscopy for the foot and hand simulations.

**Results:**

Readings were tabulated, and coloured heatmaps were generated from scatter measurements for each of the five procedures. Positions corresponding to the typical location of the surgical staff (surgeon, surgical assistant, anaesthetist, instrument (scrub) nurse, circulation (scout) nurse and anaesthetic nurse) were superimposed on heatmaps. The surgeon’s proximity to the radiation source meant this position experienced the greatest amount of radiation in all five surgical procedures. Mini C-arm doses were considered low in all procedures for positions, with and without lead protection.

**Conclusion:**

This investigation demonstrated the distribution of scattered radiation dose experienced at different positions within the orthopaedic surgical theatre. It reinforces the importance of staff increasing their distance from the primary beam where possible, reducing exposure time and increasing shielding with lead protection.

## Introduction

Intraoperative fluoroscopy has become an integral element in contemporary orthopaedic surgical practice. The ability to accurately guide reduction of fractures and anatomically localize implant placement is imperative in achieving optimal orthopaedic results [1]. Despite the benefits it serves in enhancing the technical proficiency of the surgeon, there exists a growing concern about the radiation exposure delivered to theatre personnel. Dose analytics and radiation protection are vital measures in the context of orthopaedic surgery, given the ever-increasing demand of medical radiation and the close proximity to the patient in which orthopaedic staff are positioned during cases [2]. Furthermore, a deficiency in radiological science safety training within these individuals further justifies these measures [3]. The harmful effects of radiation exposure are evident with several studies finding an increased incidence of malignant diseases in orthopaedic surgeons [4, 5]. Diverse effects such as chromosomal abnormalities and cataracts have also been reported in specialists who facilitate fluoroscopy-guided imaging procedures [6].

Staff are exposed to radiation unintentionally by X-rays being scattered when the primary beam enters the patient, changing its trajectory [7]. Understanding the behaviour of scatter radiation is central to knowing the reasoning behind radiation protection measures, thus increasing compliance and minimizing occupational risk. The effect of radiation monitoring has clear impact on clinical practice. Recently, staff involved in fluoroscopic cardiovascular procedures that demonstrated an elevated incidence of radiation‐associated lens changes prompted the International Commission on Radiological Protection (ICRP) to reduce the occupational dose limit for the eye from 150 to 20 mSv per year [8]. Several studies have accessed the dose experienced by healthcare staff in both per case and cumulative basis, particularly for cardiovascular [9], angiographic [10] and spinal [11] applications. Furthermore, mini C-arm fluoroscopy has recently become a modality of choice for surgeons who find the proposed lower radiation dose, increased mobility and ease of use appealing, though the magnitude of these doses has not been extensively explored.

The objective this study was to determine the distribution of scatter radiation from the C-arm and mini C-arm for the orthopaedic context, with particular focus on the positions of personnel and the type of orthopaedic surgery performed.

## Methods

The study was conducted in a dedicated orthopaedic operating theatre during one day in April 2019. We used a Raysafe Xi survey detector attached to a Raysafe Xi base unit. The system exhibits a dose range of 0 µSv–9999 Sv, dose rate range of 0 uSv/h–0.15 Sv/h and a maximum resolution of 0.001 µSv. Background radiation measured at ~ 100 nSv/h, though thorough pre-calibration measures were taken to ensure that background radiation was considered prior to exposure, and that the detectors were working accurately. A mobile C-arm machine (Siemens Cios Fusion Flat Panel DR; Siemens Healthcare, Erlangen, Germany) was the source of radiation for simulations of hip arthroscopy, hip replacement and knee surgery. A mini C-arm machine (Hologic Insight Fluoroscan; Hologic, Bedford, MA) produced fluoroscopy for the foot and hand surgery. The image intensifier was operated by a licenced radiographer during all exposures.

An anthropomorphic adult phantom was placed on a standard operating table to simulate the patient undergoing the procedure. The imaging source was first positioned for the hip procedures. At various angles and distances from the phantom, we recorded the scatter dose rate in microsieverts per hour (µSv/h) using consistent exposure parameters. In terms of height, the detectors were placed at the level of the phantom. Scanning parameters such as kVp, mAs, collimation and scanning time were derived from typical factors used on real patients at our institution. Typically, we took measurements at distances of 300 mm, 600 mm and 1200 mm from the phantom and in increments of 45° around the patient. Assuming an inverse square relationship, the dose rate measurements (µSv/h) were converted into (µSv/h/m2) by multiplying each measurement by the square of the distance it was measured at. Custom JavaScript code was used to complete an angle–distance grid whereby the angle range of −  180° to + 180° in 5° increments and a distance range of 300 mm to 1200 mm in 100 mm increments were implemented. Linear interpolation was then undertaken, and all values in the grid were divided by the inverse of the square of their distance to convert back to units of (µSv/h). For distances larger than 1200 mm, dose rates along the 1200 mm row using inverse square law were extrapolated. The dose rate and its corresponding Cartesian (*x*, *y*) coordinate were then uploaded to ArcGIS software (version 10.3, ESRI, California USA), where heatmaps were produced. Positions corresponding to the typical location of the surgical staff for these procedures, as advised by surgical staff at our institution, were then superimposed onto the heatmap. These positions included the surgeon, surgical assistant, anaesthetist, instrument (scrub) nurse, circulation (scout) nurse and anaesthetic nurse. We calculated the attenuation provided by the thickness of lead apron utilized in the theatre using the fitting parameters for broad-beam secondary transmission from NCRP147 in the equation published by Archer and colleagues [12]. For the C-arm, the fitting parameters for a 70 kVp beam and a 0.375-mm-thick lead apron were utilized, which produces a transmission of approximately 1.25%.

The procedure described above was then repeated to simulate knee replacement surgery using the C-arm, and foot and hand surgeries using the mini C-arm. For the knee simulation specifically, the C-arm was rotated to a lateral position, such that the beam produced was parallel to the floor. For the mini C-arm, we calculated the attenuation provided by a lead apron using the fitting parameters for a 50 kVp beam and a 0.25 mm thickness of lead, resulting in a transmission of approximately 0.5%.

## Results

**Fig. 1:**
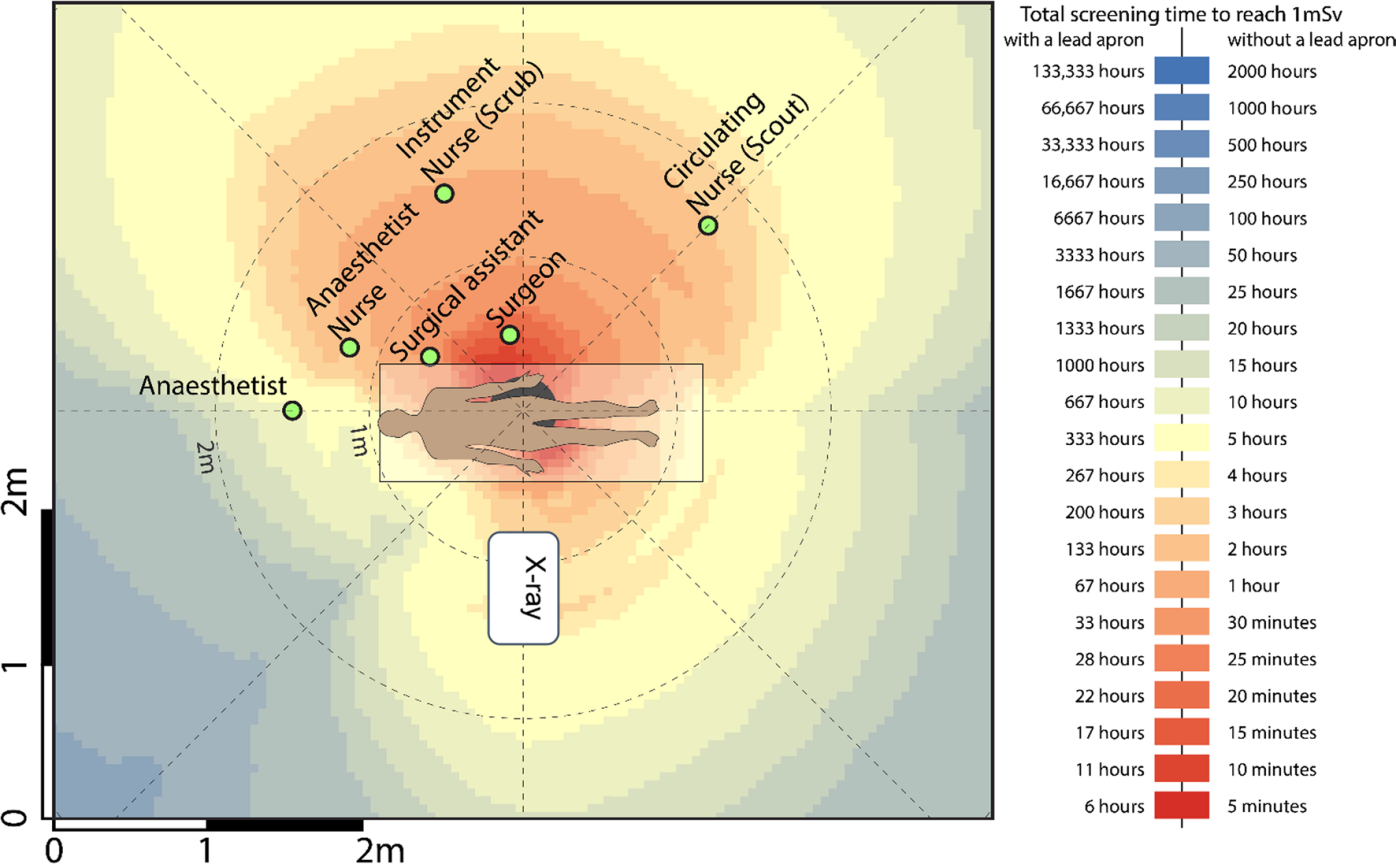
Heatmap of dose distribution for hip replacement and hip arthroscopy surgery

**Fig. 2:**
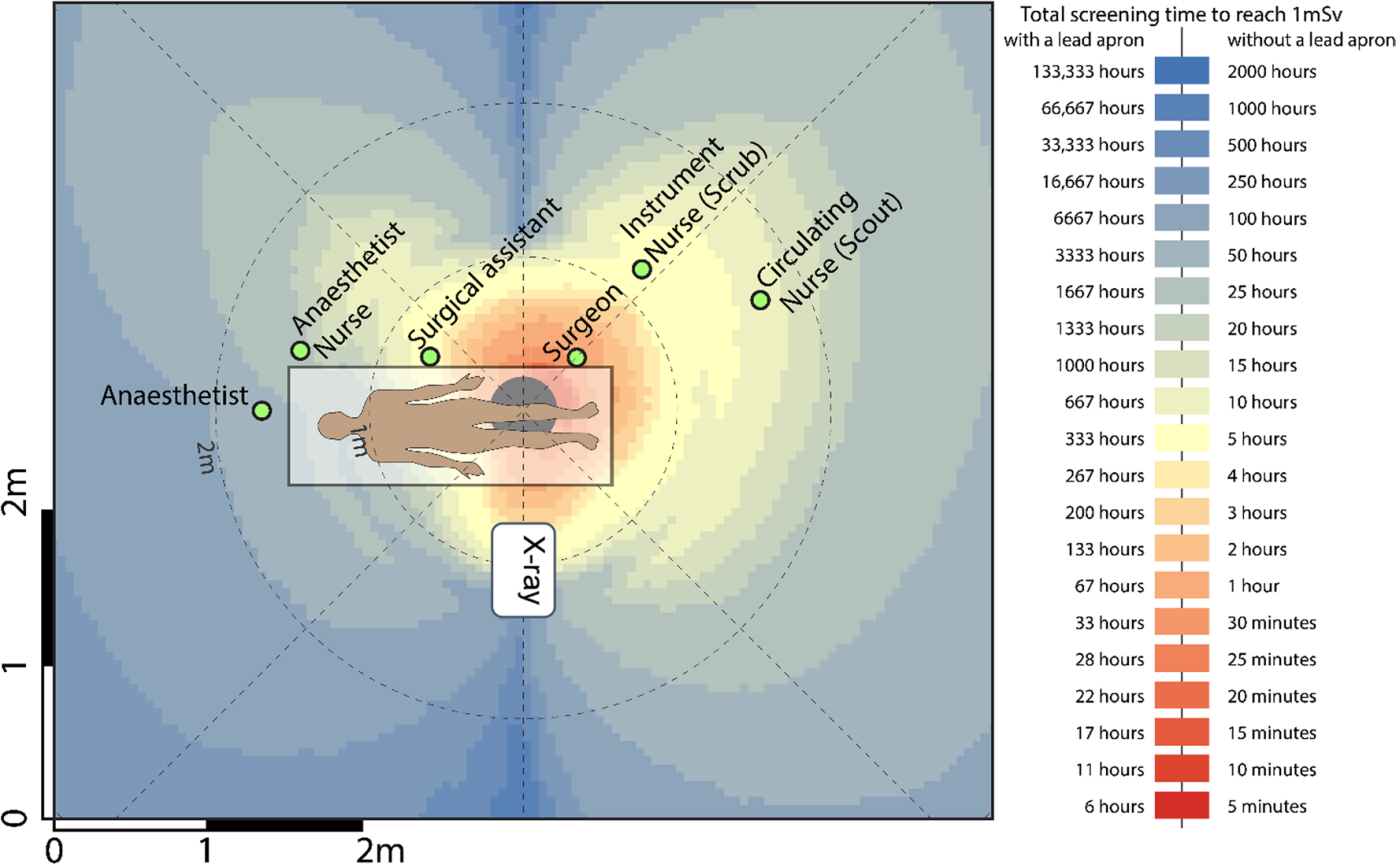
Heatmap of dose distribution for knee surgery in the lateral position

**Fig. 3:**
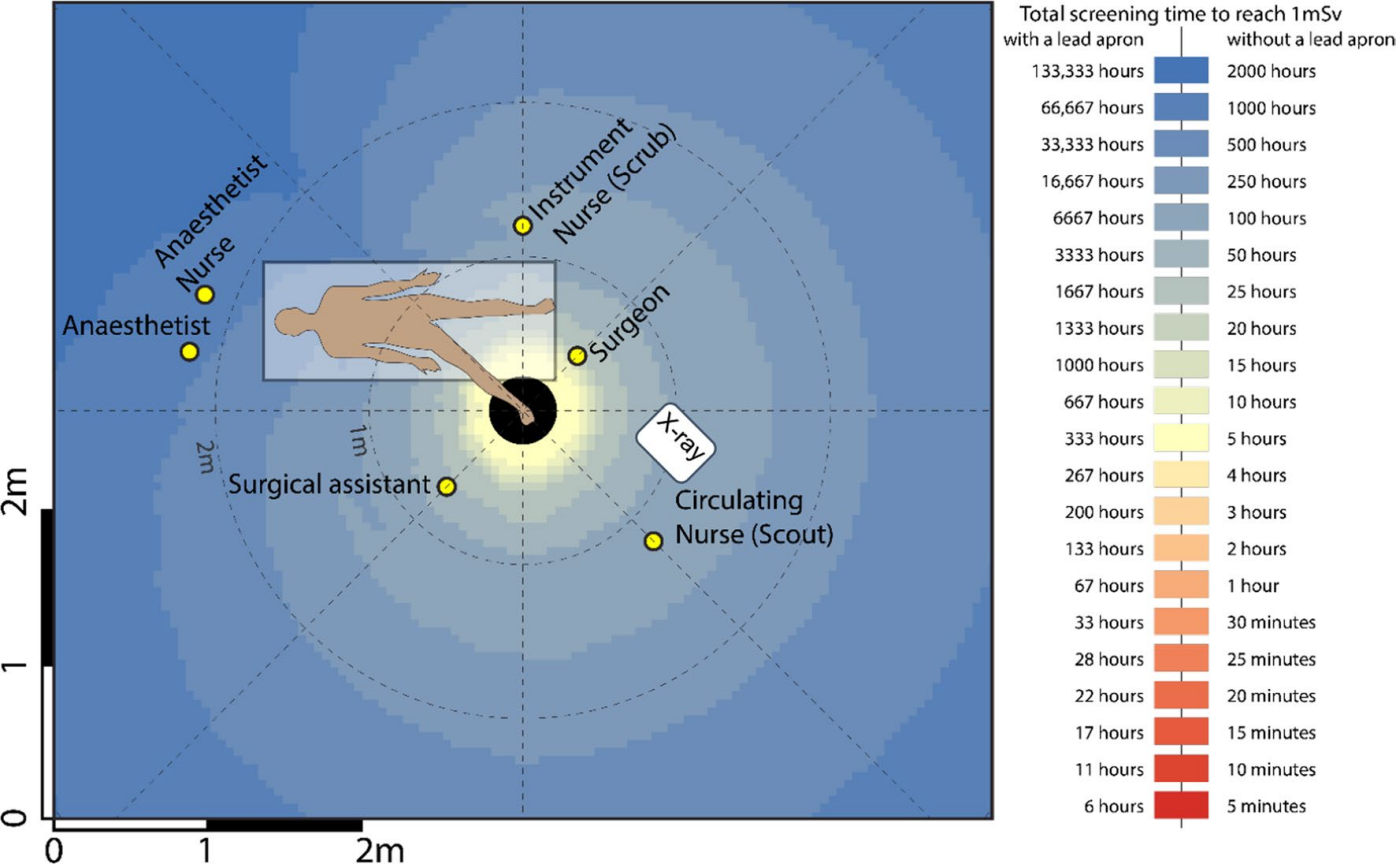
Heatmap of dose distribution for foot surgery

**Fig. 4:**
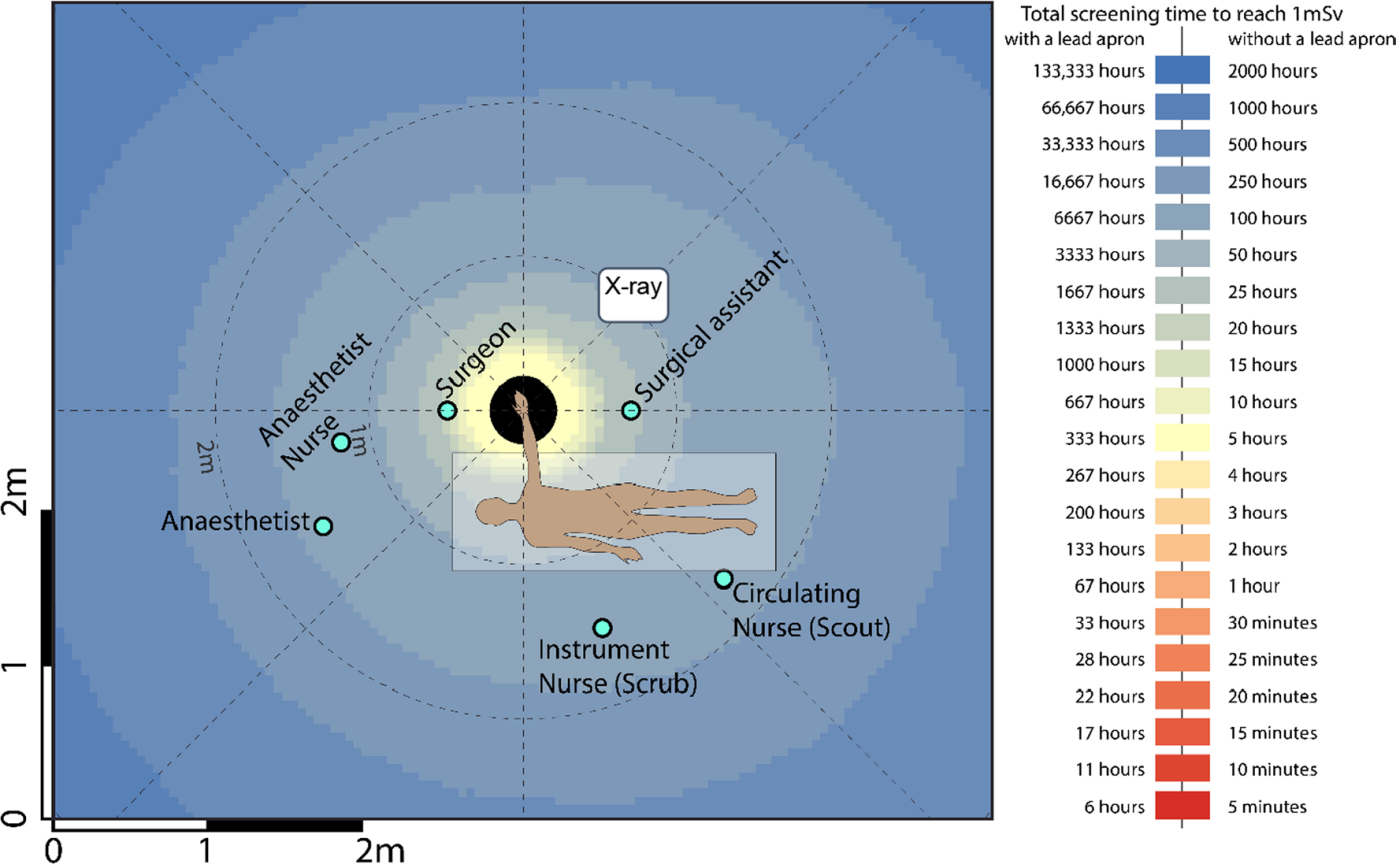
Heatmap of dose distribution for hand surgery

The highest radiation doses were measured closest to the radiation source and decreased with distance. The surgeon’s proximity to the radiation source meant this position experienced the greatest amount of radiation in all five surgical procedures. Though the surgical assistant was positioned close to the patient, the dose experienced by this position was either lower or comparable to that of the instrument and circulating nurse position in each procedure. In all procedures, the use of lead protection reduced exposure significantly, such that all roles except the surgeon and instrument in all five procedures would require greater than 10,000 cases per year to reach 1 mSv if wearing a lead apron. Mini C-arm doses were considered low in all procedures for all staff locations positions, with and without lead protection. The following tables (Tables 1, 2, 3) and heatmaps (Figs. 1, 2, 3, 4) demonstrate the dose rates at various positions around the operating theatre table for each of the five procedures.

**Table 1:** Radiation dose experienced during hip replacement and hip arthroscopy surgery

Staff member	Distance from source [m]	Dose rate (mSv/h)	Dose rate with lead apron (mSv/h)	Hip replacement		Hip arthroscopy	
				Cases per year to reach 1 mSv* No lead apron	Cases per year to reach 1 mSv* With lead apron	Cases per year to reach 1 mSv* No lead apron	Cases per year to reach 1 mSv* With lead apron
Surgeon	0.5	3.1227	0.0390	36	2870	64	5144
Surgical assistant	0.7	0.6846	0.0086	164	> 10,000	293	> 10,000
Anaesthetist	1.5	0.0453	0.0006	2472	> 10,000	4429	> 10,000
Anaesthetic nurse	1.2	0.1259	0.0016	890	> 10,000	1595	> 10,000
Instrument nurse (Scrub)	1.5	0.9080	0.0114	123	9872	221	> 10,000
Circulating nurse (Scout)	1.7	0.6984	0.0087	160	> 10,000	287	> 10,000

**Table 2:** Radiation dose experienced during knee surgery with the X-ray source in a lateral position

Staff member	Distance from source [m]	Dose rate (mSv/h)	Dose rate with lead apron (mSv/h)	Cases per year to reach 1 mSv* No lead apron	Cases per year to reach 1 mSv* With lead apron
Surgeon	0.5	1.0000	0.0125	111	8900
Surgical assistant	0.7	0.1000	0.0013	1112	> 10,000
Anaesthetist	1.7	0.0100	0.0001	> 10,000	> 10,000
Anaesthetic nurse	1.5	0.0200	0.0003	5562	> 10,000
Instrument nurse (Scrub)	1.2	0.2000	0.0025	556	> 10,000
Circulating nurse (Scout)	1.7	0.1000	0.0013	1112	> 10,000

**Table 3:** Radiation dose experienced during foot and hand surgery

Staff member	Distance from source [m]	Foot				Hand				
		Dose rate (mSv/h)	Dose rate with lead apron (mSv/h)	Cases per year to reach 1 mSv* No lead apron	Cases per year to reach 1 mSv* With lead apron	Distance from source [m]	Dose rate (mSv/h)	Dose rate with lead apron (mSv/h)	Cases per year to reach 1 mSv* No lead apron	Cases per year to reach 1 mSv* With lead apron
Surgeon	0.5	0.0462	0.0002	5033	> 10,000	0.5	0.0444	0.0002	5234	> 10,000
Surgical assistant	0.7	0.0141	0.0001	> 10,000	> 10,000	0.7	0.0234	0.0001	9913	> 10,000
Anaesthetist	2.2	0.0006	< 0.0001	> 10,000	> 10,000	1.5	0.0036	< 0.0001	> 10,000	> 10,000
Anaesthetic nurse	2.2	0.0005	< 0.0001	> 10,000	> 10,000	1.2	0.0055	< 0.0001	> 10,000	> 10,000
Instrument nurse (Scrub)	1.2	0.0040	< 0.0001	> 10,000	> 10,000	1.5	0.0047	< 0.0001	> 10,000	> 10,000
Circulating nurse (Scout)	1.2	0.0050	< 0.0001	> 10,000	> 10,000	1.7	0.0040	< 0.0001	> 10,000	> 10,000

Exposure parameters and apparatus for the two hip surgeries (replacement and arthroscopy) were identical, with the exception of exposure time.

### Hip replacement

77 kVp, 20.6 mA, 32.1 s, 0 magnification, high dose, 15 fps, 300 mm focus-to-skin distance (FSD).

### Hip arthroscopy

77 kVp, 20.6 mA, 17.9 s, 0 magnification, high dose, 15 fps, 300 mm focus-to-skin distance (FSD).

### Knee (Lateral)

60 kVp, 17 mA, 0 magnification, medium dose, 15 fps, 300 mm focus-to-skin distance (FSD).

### Foot and hand

52 kVp, 0.06 mA, 15.5 s.

## Discussion

When fluoroscopy is used in the operating theatre, the entire surgical team is exposed to scatter radiation. Scatter is produced through the interaction of the primary beam with any object, namely human tissue and equipment within the operating theatre room. The purpose of this study was to gain a more informed understanding of the distribution of scatter radiation in the orthopaedic operating theatre and the implications this has for various surgical roles.

As expected, the magnitude of scattered radiation measured was inversely proportional to the distance from the radiation source, although this was not demonstrated in a strictly radial inverse square relationship. The inverse square law is based on a point source within a vacuum, two qualities neither the patient nor the theatre possesses. Nevertheless, the theory that it is considerably safer to be on the opposite side of the patient table to the surgical team was reinforced in our study. An asymmetric distribution of stray radiation was demonstrated resulting in higher-intensity radiation on the X-ray tube side of the patient, which is likely due to the shielding supplied by the C-arm base and housing as well as the patient themselves. This leads us to suggest that the operator, and other personnel where possible, positions themselves on the machine side of the patient where possible.

This study was not without limitations. We used a specific phantom which naturally presents the inherent limitation associated with ex vivo studies, though methods including irradiation often precludes application in humans for ethical reasons. In reality, staff in theatre would contribute to both the protection of others where scatter radiation is absorbed by their bodies, or potentially be of detriment, where secondary scatter from their bodies is then projected towards their colleagues. Thus, our findings are based on the dose received by a single role without any other staff member in the room. Given that lead protection will absorb much more radiation than it scatters, however, this would therefore overestimate the dose particularly for the anaesthetic nurse, instrument and circulating nurse as they were positioned behind the surgeon and surgical assistant. Thus, our findings present a conservative dose for these positions, and their experienced doses are likely to be even lower due to shielding. In addition, positions were treated as fixed or “static” positions. Though operating theatre personnel have typical or common positions during a procedure, they are generally not static. Nevertheless, the heatmaps serve as a useful tool for determining the exposure received at any location.

## Conclusion

This investigation demonstrated the distribution of scattered radiation dose experienced at different positions within the orthopaedic surgical theatre. Dose was dependent on the region of exposed anatomy and the type of fluoroscopic machine used. Distance from the radiation source was seen to be inversely proportional to dose. Radiation doses were significantly reduced with the introduction of lead protection. Without such protection, the study shows that continual exposure particularly for the surgeon, instrument and circulating nurse, and surgical assistant positions may lead to a breach of the 1 mSv yearly limit. Mini C-arm exposures in all positions were considered low. The ICRP acknowledges that the long-term effects of any additional amounts of radiation from non-natural sources are not entirely known. Hence, as orthopaedic staff will continue to be involved with the X-ray equipment for the durations of their careers, every care should be taken to increase their distance from the primary beam where possible, reduce exposure time and increase shielding with lead protection to minimize occupational radiation.

## Data Availability

All data and materials support the published claims.
